# Comparison of granule and traditional decoction of Gancao Xiexin for cold-heat complex pattern recurrent oral ulcer: a randomized controlled trial

**DOI:** 10.3389/fphar.2025.1668985

**Published:** 2026-01-05

**Authors:** Si-Run Liu, Hai-Tao Zhu, Pei-Chi Chen, Yu-Yu Zeng, Rui-Min Wang, Hua-Chong Xu, Ze-Jian Li

**Affiliations:** 1 Hospital of Stomatology, The First Affiliated Hospital of Jinan University, Guangzhou, Guangdong, China; 2 School of Stomatology, Jinan University, Guangzhou, Guangdong, China; 3 School of Traditional Chinese Medicine, Jinan University, Guangzhou, Guangdong, China

**Keywords:** cold-heat complex syndrome, Gancao Xiexin decoction, granules, recurrent oral ulcers, traditional Chinese medicine

## Abstract

**Objective:**

To compare the clinical efficacy and metabolites of Gancao Xiexin Decoction (GXD) granules versus traditional decoction in patients with recurrent oral ulcers (ROU) and cold-heat complex syndrome.

**Methods:**

A randomized trial of 79 patients evaluated the clinical efficacy of both formulations for recurrent oral ulcers (ROU) with cold-heat complex syndrome. HPLC-ICP-MS was used to analyze metabolites and heavy metals.

**Results:**

The Traditional Chinese Medicine (TCM) group, granule group and biomedicine group all showed significant improvements in clinical efficacy, inflammatory factors, and immune markers (*P* < 0.05). However, no significant differences were found between granules and decoction in salivary inflammatory factors, immune indicators, pain VAS scores, or TCM syndrome scores (*P* > 0.05). HPLC-ICP-MS analysis showed 60.4% common peak ratio in decoction and 68.1% in granules, with significantly lower heavy metal levels (Cu, As, Cd, Hg, Pb) in granules (*P* < 0.01).

**Conclusion:**

GXD granules and traditional decoction showed comparable clinical efficacy in managing ROU with cold-heat complex syndrome. Notably, granule formulations exhibited lower heavy metal content, suggesting potential advantages in safety profile.

## 1 Introduction

Recurrent oral ulcer (ROU) is a self-limiting mucosal lesion of the oral cavity characterized by periodic recurrences, clinically presenting as recurrent ulcerative lesions (Sánchez-Bernal et al., 2020a; Gasmi Benahmed et al., 2021; Spano et al., 2022; Milia et al., 2022). These ulcerations often inducing lancinating pain, impairing eating/speech, and significantly reducing quality of life (Milia et al., 2022; Conejero Del Mazo et al., 2023; Zhao et al., 2025). In recent years, with changes in lifestyle and dietary patterns as well as increased psychosocial stress among the Chinese population, the incidence of this condition has been rising annually (Sánchez-Bernal et al., 2020b; Elias et al., 2022).

Current clinical management of ROU focuses on acute-phase anti-inflammatory control, expediting mucosal healing, and shortening ulcer duration in the short term, while prioritizing immune microenvironment modulation to reduce recurrence risk in the long term (Sánchez-Bernal et al., 2020a; Gasmi Benahmed et al., 2021; Lau and Smith, 2022; Parra-Moreno et al., 2023). However, existing treatments primarily relying on physical approaches and topical medications remain suboptimal, exhibiting high recurrence rates. Recent years have witnessed the growing recognition of traditional Chinese medicine (TCM) in treating ROU, with its distinctive approach of addressing both root and branch causes while integrating systemic and local therapies, effectively promoting ulcer healing and reducing recurrence rates. Within the TCM therapeutic framework, recurrent aphthous ulcers are referred to as “Huhuo disease” in the classic medical text *Jin Gui Yao Lue* (*Essentials from the Golden Cabinet*), which describes the condition as presenting with symptoms resembling typhoid fever. For which Gancao Xiexin Decoction (GXD) is recommended as the primary treatment (Chen et al., 2016). The *Treatise on Cold Damage* records that GXD, originally formulated by Zhang zhongjing—the revered physician of the late Eastern Han dynasty—follows the therapeutic principle of “benefiting qi and harmonizing the stomach, resolving masses and stopping vomiting” (Zhang et al., 2023). Rooted in the regulatory concepts of harmonizing pivot mechanisms and balancing pungent-opening with bitter-descending actions, this formula demonstrates unique mucosal repair-regulating effects in conditions characterized by middle-jiao cold-heat complex and disordered ascending-descending functions. Chinese herbal granules represent a modern pharmaceutical preparation derived from traditional medicinal materials through advanced extraction, purification, and concentration technologies, resulting in convenient granular dosage forms for clinical use (Wang et al., 2021). Their popularity has been steadily increasing due to advantages such as easy storage and administration without the need for decoction.

This study aims to analyze and compare the impurity profiles and heavy metal contents of GXD granules versus traditional herbal decoctions using High-Performance Liquid Chromatography coupled with Inductively Coupled Plasma Mass Spectrometry (HPLC-ICP-MS). Additionally, a clinical trial will be conducted to evaluate the therapeutic efficacy of both formulations in treating ROU of the cold-heat complex syndrome, with a focus on assessing clinical equivalence and impurity levels between the two dosage forms.

## 2 Materials and methods

### 2.1 Untargeted metabolomics analysis

#### 2.1.1 Sample preparation

The Gancao Xiexin Decoction (GXD) used in this study was prepared according to the classic prescription recorded in *Treatise on Cold Damage*. All crude botanical drugs were purchased from [Guangdong EFORNG PHARMACEUTICAL Co., Ltd, Origin: China] and met the quality standards of the Chinese Pharmacopoeia (2020 edition).

The botanical composition is detailed in Table 1. The mixed drugs were soaked in 2 L of drinking water for 30 min, then decocted by bringing to a boil and simmering for 40 min. The decoction was filtered. The residue was added to 1.5 L of water and decocted again for 30 min. The two resulting decoctions were combined and concentrated to a final volume of 400 mL. The prepared GXD was stored at 4 °C until use.

**TABLE 1:** Botanical composition and identification of Gancao Xiexin Decoction.

Chinese name	Dosage	Botanical drug name	Plant source [family; pharmacopoeial drug name]
Sheng Gancao	12g	Glycyrrhizae Radix et Rhizoma	Glycyrrhiza uralensisFisch. ex DC. [Fabaceae; Glycyrrhizae radix et rhizoma]
Huangqin	9g	Scutellariae Radix	Scutellaria baicalensisGeorgi [Lamiaceae; Scutellariae radix]
Huanglian	3g	Coptidis Rhizoma	Coptis chinensisFranch. [Ranunculaceae; Coptidis rhizoma]
Ganjiang	9g	Zingiberis Rhizoma	ZingiberofficinaleRoscoe [Zingiberaceae; Zingiberis rhizoma]
Banxia	9g	Pinelliae Rhizoma	Pinellia ternata (Thunb.) Breit. [Araceae; Pinelliae rhizoma]
Dazao	12pieces (∼36 g)	Jujubae Fructus	Ziziphus jujubaMill. [Rhamnaceae; Jujubae fructus]

We took 100 μL of the decoction. This was mixed with 300 µL methanol, vortexed for 10 min, centrifuged at 13,000 rpm for 10 min, and the supernatant was analyzed by HPLC. For GXD granules (batch number Q2401007, manufactured by Guangdong EFORNG PHARMACEUTICAL Co., Ltd.), 13.5 g of granules were dissolved in 40 mL hot water, shaken to homogeneity, and sonicated for 30 min. Then, 100 µL of the solution was mixed with 300 µL methanol, vortexed for 10 min, centrifuged at 13,000 rpm for 10 min, and the supernatant was analyzed by HPLC.

#### 2.1.2 Instrumentation

Centrifuges: D3024R refrigerated centrifuge (Beijing Dason Technology). Vortex mixer: MX-F vortex oscillator (Wuhan Servicebio Technology). Ultrasonic cleaner: JP-040S ultrasonic cleaner (Shenzhen Jiemeng Equipment). Autosampler: Direct-Q®5 ultrapure water system (Merck Millipore, Germany). Chromatography system: UltiMate 3000 RS UHPLC (Thermo Fisher Scientific). Mass spectrometer: Q Exactive™ HF Orbitrap mass spectrometer (Thermo Fisher Scientific)

#### 2.1.3 Data acquisition

Data acquisition was performed using ultra-high-performance liquid chromatography coupled with quadrupole-orbitrap high-resolution mass spectrometry (UHPLC-Q-Orbitrap HRMS), with parameters optimized for comprehensive metabolite profiling. Mass spectrometry conditions​ included electrospray ionization (ESI) in positive/negative switching mode, full MS/dd-MS^2^ scan mode (full scan range: 100–1,500 m/z), resolution settings of 70,000 (full MS) and 17,500 (dd-MS^2^), and stepped collision energy (30, 40, 60 eV). The capillary temperature was maintained at 300 °C, with sheath gas (40 arb nitrogen) and auxiliary gas (15 arb nitrogen, 350 °C) to ensure stable ion transmission. Chromatographic conditions employed an AQ-C18 column (150 × 2.1 mm, 1.8 μm, Welch Materials) maintained at 35 °C, with a mobile phase consisting of 0.1% formic acid in water (A) and acetonitrile (B). A linear gradient elution program was applied over 30 min: 0–5 min (98% A, 2% B, initial isocratic hold), 5–10 min (linear decrease to 80% A, 20% B), 10–15 min (adjustment to 50% A, 50% B), 15–20 min (rapid change to 20% A, 80% B), 20–27 min (maintenance at 5% A, 95% B), 27–28 min (return to 98% A, 2% B), and 28–30 min (equilibration at initial conditions). The flow rate was 0.3 mL/min, with an injection volume of 5 μL. Raw data were acquired for 30 min per sample to ensure complete elution of metabolites.

#### 2.1.4 Processing

Raw data were processed using Compound Discoverer 3.3 (Thermo Fisher Scientific) for peak alignment, normalization, and identification. Metabolite annotation was conducted via mzCloud database (mzCloud score ≥60) with the following criteria: (a)Molecular formula prediction using isotopic distribution patterns; (b)MS/MS fragmentation pattern matching; (c)Retention time (RT) alignment across samples.

Instrument stability was monitored using a pooled quality control (QC) sample every 5 injections. Data normalization was performed using sum normalization to account for sample concentration variations.

### 2.2 Clinical trial

#### 2.2.1 Participants and inclusion criteria

This study enrolled patients with ROU of the cold-heat complex syndrome for clinical evaluation. Healthy volunteers and patients meeting the inclusion criteria were recruited from the Department of Stomatology at the First Affiliated Hospital of Jinan University.

Participants must meet the following criteria: aged 25–55 years; fulfilled the aforementioned diagnostic criteria for both TCM syndromes and biomedicine diseases; have a documented history of at least two ROU episodes, with disease duration exceeding 1 year or ulcer recurrence at least monthly; and provide written informed consent after full understanding of the study protocol. Exclusion criteria comprised: patients aged <25 or ≥55 years; obesity (BMI >28), pregnancy, or lactation; concurrent use of medications that might interfere with efficacy evaluation. Dropout criteria included: voluntary withdrawal by the patient; loss to follow-up; poor compliance; or study discontinuation mandated by the investigator due to complicating conditions.

#### 2.2.2 Diagnostic criteria and study participation requirements for patients with cold-heat complex syndrome ROU

The Biomedicine diagnostic criteria for ROU primarily rely on characteristic clinical history (recurrent, periodic, and self-limiting nature) and physical findings. Patients generally present with recurrent round or oval ulcers, which have the clinical characteristics of “yellow, red, concave, and painful”, that is, the ulcer surface is covered with a yellow false membrane, surrounded by a red halo, with a central depression and obvious pain.

Diagnostic criteria for TCM syndrome type (developed with reference to Guidelines for Clinical Research of New Traditional Chinese Medicine Drugs [Trial] (Zheng, 2002)): The primary symptoms include “pale-red oral ulcers, dull pain, and recurrent episodes”; secondary symptoms comprise “bitter taste in mouth, dry mouth, epigastric fullness, postprandial abdominal distension, fatigue, red tongue, enlarged tongue body, yellow greasy or white greasy coating, and soggy or rapid pulse.” A diagnosis is confirmed when the primary symptoms are present alongside at least two secondary symptoms.

#### 2.2.3 Clinical trial design and sample collection

This study employed a randomized clinical trial design comparing the therapeutic effects of GXD decoction, GXD granules, and Biomedicine. To evaluate the treatment efficacy in patients with cold-heat complex syndrome ROU, we compared baseline data between healthy controls and patient groups, with ROU patients subsequently randomized into three intervention arms: decoction group, granule group, and Biomedicine group. The GXD decoction group received one dose daily, prepared by decocting the herbs twice to yield 400 mL of liquid, administered in two equal portions (morning and evening) via oral retention (holding the solution in the mouth for 2–3 min before swallowing), with each treatment course lasting 7 days. The Chinese herbal granule group followed the same oral retention protocol (2–3 min before swallowing), taking the granules dissolved in water twice daily (morning and evening) over 7 days per course. The Biomedicine control group used a 0.02% chlorhexidine acetate gargle solution for oral rinsing 3–5 times daily, supplemented with oral vitamin B_2_ (20 mg three times daily) and vitamin C (100 mg three times daily), also over a 7-day treatment course.

Saliva samples (3 mL) were collected from all participants (both healthy controls and ROU patients) before and after treatment, following a 2-h fasting period. Samples were centrifuged at 2,000 rpm for 10 min to separate the supernatant, which was then analyzed for immune factors using enzyme-linked immunosorbent assay (ELISA). Inflammatory cytokines in saliva were quantified via Luminex xMAP liquid chip technology (Merck Millipore, United States), with absolute cytokine concentrations determined using the Milliplex Analyst software platform.

#### 2.2.4 Clinical efficacy assessment

TCM syndrome scoring was conducted according to the *Standards for Diagnosis and Therapeutic Evaluation of Traditional Chinese Medicine Diseases and Syndromes* (National Administration of Traditional Chinese Medicine, 1994) and the Guidelines for Clinical Research of New Traditional Chinese Medicines (Trial) (Zheng, 2002). A TCM syndrome scoring system was established using a ordinal variable methodology, with assessments performed twice—before treatment initiation and at the end of the treatment course.

The primary symptoms (pale-red ulcers, dull pain, recurrent episodes) were graded on a scale of 0–6 (0 = absent, 2 = mild, 4 = moderate, 6 = severe), while secondary symptoms (bitter taste/dry mouth, epigastric stuffiness, postprandial abdominal distension, fatigue) were scored on a scale of 0–3 (0 = absent, 1 = mild, 2 = moderate, 3 = severe).

Following the Guidelines for Clinical Research of New Traditional Chinese Medicines (Trial) (Zheng, 2002), therapeutic outcomes were classified into four categories: clinical cure, marked efficacy, efficacy, and inefficacy. Clinical cure was defined as complete disappearance or near-total resolution of clinical symptoms, with a ≥95% reduction in syndrome score and normalization of laboratory parameters. Marked efficacy indicated significant clinical improvement (≥70% but <95% reduction in syndrome score) with notable laboratory parameter improvements. Efficacy required some symptomatic improvement (≥30% but <70% reduction in syndrome score) accompanied by measurable laboratory parameter changes. Inefficacy was defined as no symptomatic improvement or worsening (<30% reduction in syndrome score) without laboratory parameter benefits. Evaluations were performed twice—before treatment initiation and at the end of the treatment course—to assess therapeutic effects comprehensively.

Pain intensity was evaluated using a 10-point Visual Analog Scale (VAS), with assessments conducted twice—once before treatment initiation and immediately after the completion of the treatment course.

#### 2.2.5 Statistical analysis

Statistical analyses were performed using SPSS 26.0 software. Categorical variables were expressed as percentages (%) and compared using the chi-square test. Continuous variables were presented as mean ± standard deviation (x ± s), with comparisons between groups conducted using the t-test for normally distributed data and the Wilcoxon rank-sum test for non-normally distributed data. For comparisons within the same group (e.g., pre-vs. post-intervention), the paired samples *t*-test was used for normally distributed data, while the Wilcoxon signed-rank test was employed for non-normally distributed data. A *p*-value of less than 0.05 was considered statistically significant.

#### 2.2.6 Ethical considerations and participant rights

This study was conducted in strict adherence to the Declaration of Helsinki (2013) and the Guidelines for Good Clinical Practice (GCP). Ethical approval was obtained from the Institutional Review Board (IRB) of Jinan University Affiliated Hospital (Approval No.: KY-2023-333) prior to participant enrollment. Written consent was obtained from all participants after explaining the study’s purpose, procedures, risks (e.g., herbal allergy, bitter taste), and benefits (e.g., symptom relief). Participant data (demographics, clinical outcomes, biomarker results) were de-identified and stored on encrypted servers accessible only to the research team. Publication of results will not disclose personal identifiers.

## 3 Results

### 3.1 HPLC analysis results

**FIGURE 1:**
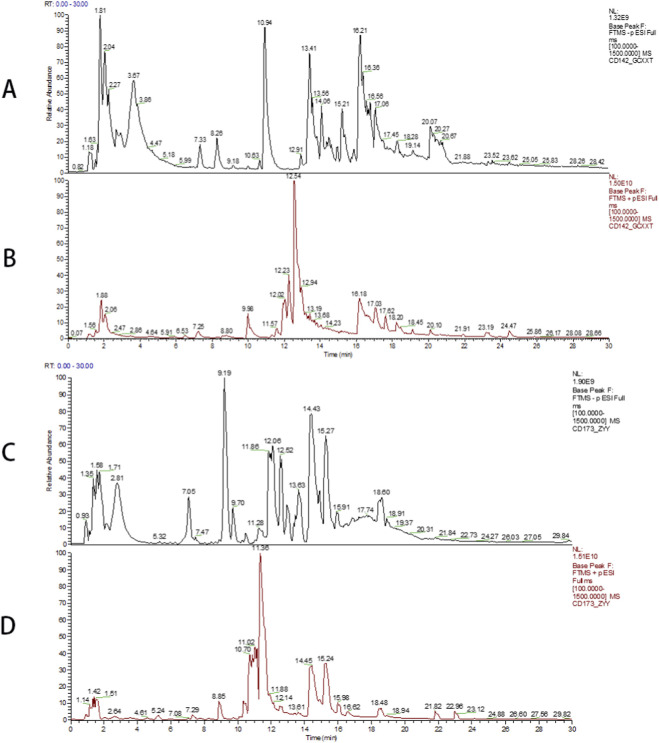
Total Ion current chromatogram. **(A)** Negative ion mode total ion current chromatogram of granules; **(B)** Positive ion mode total ion current chromatogram of granules; **(C)** Negative ion mode total ion current chromatogram of traditional decoction; **(D)** Positive ion mode total ion current chromatogram of traditional decoction.

HPLC analysis identified 459 metabolites in the GXD decoction under positive ion mode and 436 metabolites in the granule formulation, while 261 metabolites were detected in the decoction under negative ion mode compared to 216 metabolites in the granules (Figure 1). The identified metabolites were matched against the mzCloud database using CD3.3 software, with a matching score threshold of 95 applied. This analysis revealed 108 distinct peaks in the decoction and 94 peaks in the granule formulation, with 64 shared peaks between the two dosage forms. The decoction exhibited a 60.4% proportion of shared peaks, while the granules demonstrated a higher 68.1% consistency in peak profiles.

### 3.2 Comparative analysis of metabolites profiles between dosage forms

**FIGURE 2:**
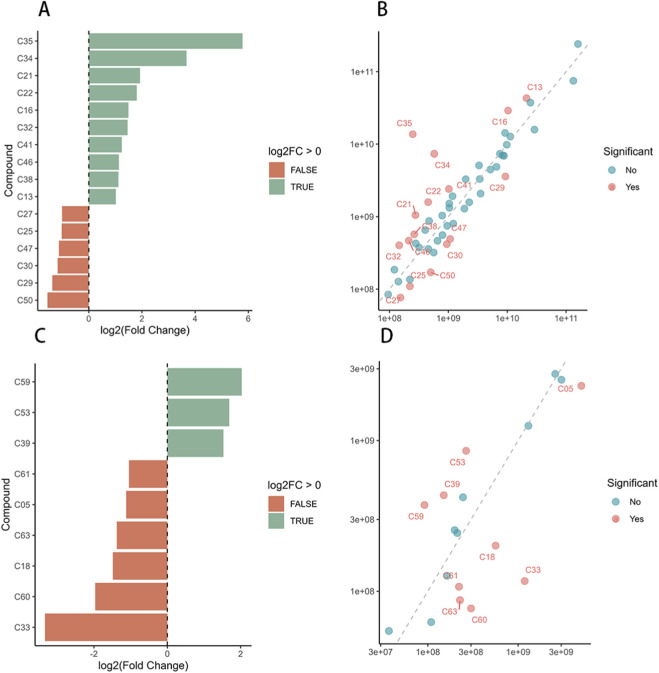
Bar plots and scatter plots of differentially abundant components. **(A)** Positive ion mode bar plot; **(B)** Positive ion mode scatter plot; **(C)** Negative ion mode bar plot; **(D)** Negative ion mode scatter plot. DCT: traditional decoction n group; GR: granule group; Bar plots display the most significantly different compounds (ranked by |log2 FC|), with red indicating components more abundant in the traditional decoction group and green representing those more abundant in the granule group.

Data processing was performed using R software. For data standardization, raw peak areas from both GXD decoction and granule formulations underwent sum-normalization to eliminate variations in total ion intensity between samples. The fold change (FC) between granules and decoction was calculated as FC = granule peak area/decoction peak area, followed by log2 transformation to obtain log2FC values. Metabolites with significant differences were identified using the threshold |log2FC| > 1 (equivalent to FC > 2 or FC < 0.5). Visualization of differentially expressed metabolites was performed using ggplot2 in R, generating bar plots and scatter plots (Figure 2). In negative ion scanning, the content of apigetrin in the decoction was over eight times higher than that in the granules, while chrysin was approximately fourfold more abundant in the decoction; conversely, methylsuccinic acid was present at only one-quarter the level in the decoction compared to the granules. In positive ion scanning, the content of 18β-glycyrrhetinic acid was approximately 55-fold higher in the granules than in the decoction, while shogaol was about 12-fold more abundant in the granules. A scatter plot of log_10_-transformed normalized peak areas revealed that most metabolites clustered closely along the y = x line, indicating strong overall correlation in chemical composition between the granules and decoction (represented by blue dots). Outliers (marked as red dots) deviated from the y = x line, with red points located above the line indicating higher concentrations in the granules and those below signifying greater abundance in the decoction. Significantly differential metabolites (log_2_FC > 1 or < −1) between the two formulations were specifically labeled.

### 3.3 Heavy metal detection by ICP-MS

**FIGURE 3:**
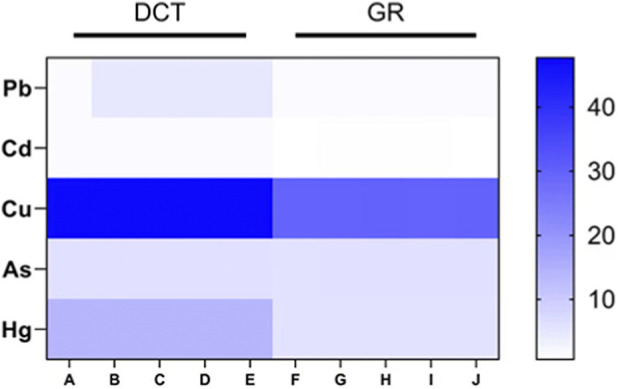
Heatmap of heavy metal element detection. DCT: Traditional decoction group; GR: Granule group; *: *P* < 0.001.

ICP-MS was used to Pb, Cd, Cu, As, and Hg in both the decoction and granule formulations of GXD. As shown in Figure 3, the decoction contained significantly higher levels of all five heavy metals compared to the granules (*P < 0.001*).

### 3.4 Clinical trial results

#### 3.4.1 Baseline comparison

**FIGURE 4:**
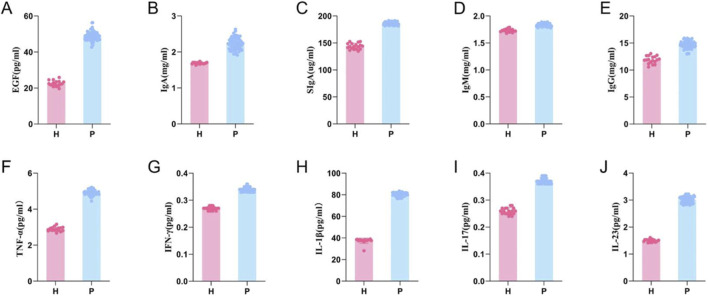
Comparison between healthy group and patient group before intervention. Detection of cytokines levels including EGF **(A)**, IgA **(B)**, SIgA **(C)**, IgM **(D)**, IgG **(E)**, TNF-α **(F)**, INF-γ **(G)**, IL-1β **(H)**, IL-17 **(I)**, IL-23 **(J)**. The ordinate represents the expression level of the factor, while the abscissa indicates the experimental groups. H: Healthy group; P: Patient group.

This study enrolled 79 patients with cold-heat complex syndrome ROU, who were randomly assigned to three groups: 51 in the TCM group (25 in the decoction subgroup and 26 in the granule subgroup) and 28 in the biomedicine group. Additionally, 18 healthy participants without ROU were recruited (these individuals were not included in post-treatment comparisons). Comparison of pre-treatment biomarker levels between healthy controls and ROU patients (Figure 4; Supplementary Table S1) revealed statistically significant differences in both inflammatory and immune factor levels (*P < 0.001*).

#### 3.4.2 Comparative clinical efficacy of GXD (decoction and granule formulations) vs. biomedicine

**FIGURE 5:**
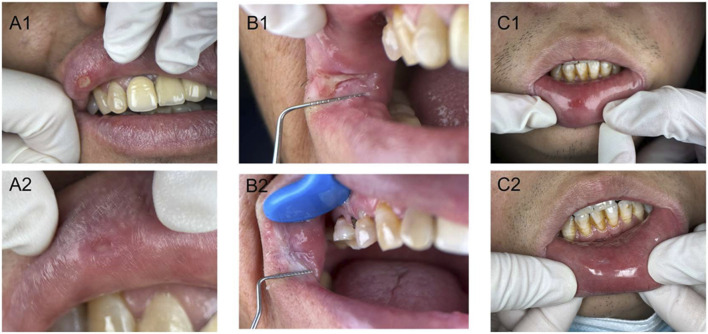
Clinical photographs of patients with cold-heat complex recurrent oral ulcers before and after treatment in three groups. A1, Pre-treatment in granule group; A2, Post-treatment in granule group; B1, Pre-treatment in traditional decoction group; B2, Post-treatment in traditional decoction group; C1, Pre-treatment in biomedicine group; C2, Post-treatment in biomedicine group.

**FIGURE 6:**
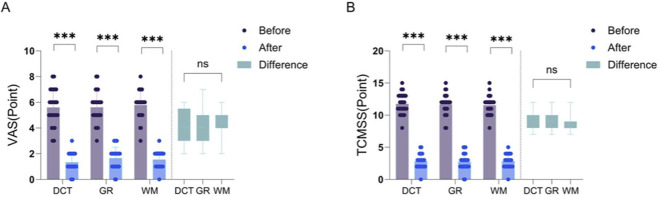
Statistical analysis of VAS pain scores **(A)** and TCM syndrome scores (TCMSS) **(B)** before and after treatment in three patient groups. The ordinate represents the expression level of the factor, while the abscissa indicates the experimental groups. The left side of the dashed line shows within-group comparisons, while the right side shows between-group comparisons. DCT: traditional decoction group; GR: granule group; WM: biomedicine group; Before: Factor expression level in saliva before treatment; After: Factor expression level in saliva after treatment; Difference: Difference in factor expression level before and after treatment; *: P < 0.05; **: 0.001 < P < 0.01; ***: P < 0.001; ns: P > 0.05.

We have displayed some clinical pictures in Figure 5. All three treatment groups demonstrated significant clinical improvement, with oral ulcers achieving substantial healing within a week post-treatment. Comparison of clinical outcome measures before and after GXD treatment in patients with cold-heat complex syndrome ROU revealed statistically significant reductions in both VAS pain scores and TCM syndrome scores within all three groups (*P < 0.001*). However, comparison of the magnitude of these changes between groups showed no statistically significant differences (*P > 0.05*), as illustrated in Figure 6.

Clinical efficacy evaluation (Table 2) showed the following outcomes: for the decoction group, the recovered rate was 4% (1/25), marked effect 68% (17/25), and effective 28% (7/25); for the granule group, 7.7% (2/26), 69.2% (18/26), and 23.1% (6/25) respectively; and for the biomedicine group, 3.6% (1/28), 67.9% (19/28), and 28.6% (8/28). Statistical analysis revealed no significant difference in overall efficacy among the three groups (*P = 0.976*).

**TABLE 2:** Clinical efficacy evaluation.

Group	Total	Recovered [Cases (%)]	Marked effect [Cases (%)]	Effective [Cases (%)]	Chi-square test	
					X²	P
					Value	Value
Decoction group	25	1 (4%)	17 (68%)	7 (28%)	​	​
Granule group	26	2 (7.7%)	18 (69.2%)	6 (23.1%)	0.947	0.976
Biomedicine group	28	1 (3.6%)	19 (67.9%)	8 (28.6%)	​	​

**FIGURE 7:**
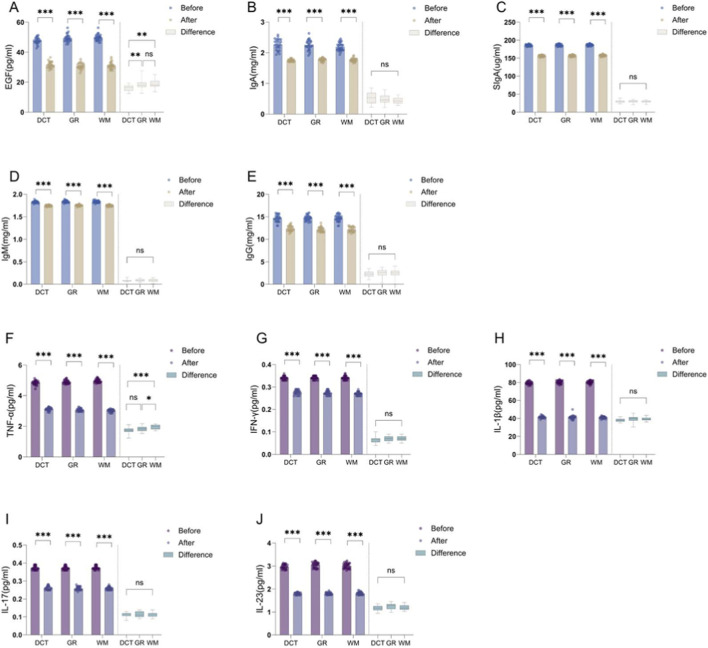
Differential expression of cytokines in patients across three groups before and after treatment. These figures show the expression levels of cytokines, including EGF**(A)**, IgA**(B)**, SIgA**(C)**, IgM**(D)**, IgG**(E)**, TNF-α**(F)**, INF-γ**(G)**, IL-1β**(H)**, IL-17**(I)**, IL-23 **(J)** before and after the treatment with TCM or biomedicine. The vertical axis represents the level or score of the factor, and the horizontal axis represents the groups. To the left of the dotted line indicates the within-group comparison, and to the right of the dotted line indicates the between-group comparison; DCT: traditional decoction group; GR: granules group; WM: biomedicine group; Before: Before the patient receives treatment; After: After the patient receives treatment; Difference: The difference in the expression level of the factor before and after treatment; *: *P < 0.05*; **: *0.001 < P < 0.01*; ***: *P < 0.001*; ns: *P > 0.05*.

Analysis of cytokine changes (Figure 7; Supplementary Table S2) before and after treatment in all three groups (EGF, IgA, IgM, IgG, SIgA, TNF-α, IFN-γ, IL-1β, IL-7, IL-23) demonstrated statistically significant differences (*P < 0.001*) regardless of whether patients received decoction, granules, or biomedicine for ROU treatment, subsequent ANOVA analysis of the treatment-induced changes revealed no statistically significant differences (*P > 0.05*) for IgA, IgM, IgG, SIgA, IFN-γ, IL-1β, IL-7, and IL-23 among the three groups, while EGF and TNF-α showed significant differences (*P < 0.05*).

## 4 Discussion

Originating from the *Treatise on Cold Damage Diseases*, the classic compound prescription GXD is based on the theoretical framework of harmonizing cold and heat and fortifying the middle jiao (Liu et al., 2021). It primarily addresses digestive system disorders accompanied by symptoms such as epigastric fullness and rigidity, diarrhea with loose stools, vomiting, nausea, and mental distress. The key points for pattern identification include a soggy and weak pulse, a pale red tongue with a white, greasy, and slightly yellow coating. By rectifying the condition of mixed deficiency and excess resulting from the dysfunction of the middle jiao’s ascending and descending movements, it demonstrates significant regulatory effects in treating gastrointestinal dysfunction characterized by the intermingling of cold and heat (Liu et al., 2021). Zhang et al. conducted a meta-analysis on the efficacy of modified GXD in treating RAU. The results demonstrated that the modified GXD was significantly superior to conventional Biomedicine in the treatment of ROU (Zhang et al., 2021). Recent pharmacological analyses reveal that the active metabolites of this formula exhibit definitive anti-inflammatory effects and gastrointestinal mucosal protective properties. These multifaceted biological activities may elucidate the modern scientific basis of its “treating different diseases with the same method” theory, providing crucial research directions for deciphering the mechanisms underlying classic herbal formulas (Xu et al., 2012).

Although ROU are closely associated with immune dysregulation and a localized inflammatory microenvironment, the precise pathogenesis remains incompletely understood. Xiang et al. demonstrated that altered IL-17 expression levels are significantly associated with ROU susceptibility (Xiang et al., 2020). Th17 cells, a subset of CD4^+^ T cells, require antigen stimulation and activation of antigen-presenting cells (APCs) during their differentiation through the Th17 differentiation pathway. These cells produce interleukin-17 (IL-17), which mediates immune responses involving epithelial cells and neutrophils to defend against microbial pathogens while also contributing to the pathogenesis of autoimmune diseases (Maddur et al., 2012; Shao et al., 2020; Wang et al., 2022). Kemal Ozyurt et al. (Ozyurt et al., 2014) demonstrated significantly elevated IL-17 levels in recurrent aphthous ulcer patients compared to healthy controls (*P < 0.05*). The host primarily utilizes IL-17 to defend against infections from *Staphylococcus* epidermidis, Rodentibacter pneumotropicus, and *Candida* albicans in the oral cavity, suggesting that Th17 cells play a crucial role in inflammatory processes, mucosal surface inflammation, and host defense against bacterial and fungal pathogens (Zhu and Qian, 2012; Fang et al., 2024). Previous studies have reported conflicting associations between TNF-α and ROU, with early research failing to detect this cytokine in patient serum. However, Zhang Suxin et al. found significantly elevated salivary TNF-α concentrations in ROU patients during both active and remission phases compared to healthy controls *(P < 0.05*) (Zhang et al, 2016a). Animal studies have demonstrated that GXD can regulate intestinal microbiota dysbiosis in mice, with researchers hypothesizing that this effect is mediated through SIgA expression (Guo et al., 2019). Additionally, a meta-analysis of 10 case-control studies (including 884 recurrent aphthous ulcer patients and 1,104 control participants) revealed that IL-1β gene mutations may increase susceptibility to recurrent aphthous ulcers (Wu et al., 2018). Based on these findings, we hypothesize that altered IL-1β expression levels might similarly influence the pathogenesis of recurrent oral ulcers.

Liquid chromatography analysis of GXD metabolites revealed that the decoction contained 60.4% shared peaks, while the granule formulation showed 68.1% shared peaks. These results indicate that compared to the granules, the decoction has a more complex composition, suggesting that the industrialized granule preparation effectively eliminates certain non-shared impurity peaks through its purification process. ICP-MS analysis revealed significantly lower levels of Cu, As, Cd, Hg, and Pb in GXD granules compared to the decoction (*P < 0.01*), a difference with important clinical implications. Research indicates that due to the bioaccumulative properties of heavy metals, prolonged exposure can induce renal and bladder abnormalities, ultimately causing irreversible damage to human health (Teschke, 2024; Khalaf et al., 2024). The granule manufacturing process (including high-temperature sterilization and excipient adsorption) effectively reduces total heavy metal content, thereby minimizing hepatic and renal accumulation risks. For patients requiring continuous management of chronic recurrent oral ulcers, the improved impurity levels of granules supports their selection as a viable option for long-term therapy.

Human saliva, secreted by the salivary glands within the oral cavity, is a specialized biological fluid composed primarily of 94%–99% water, 0.5% organic substances, and 0.2% inorganic metabolites (Zhang et al, 2016b). As a critical regulator of oral homeostasis, saliva facilitates essential functions including food digestion, mucosal cleansing, and lubrication (Helmerhorst and Oppenheim, 2007). In addition to secretions from salivary glands, saliva contains food debris, serum metabolites, oral microorganisms and their metabolic byproducts, leukocytes, and desquamated epithelial cells. Tenovuo et al. demonstrated that saliva contains abundant antimicrobial proteins (Tenovuo, 2002). Based on these findings, this study measured five inflammatory cytokines (TNF-α, IFN-γ, IL-1β, IL-17, IL-23) and five immune-related factors (EGF, IgA, IgM, IgG, sIgA) in saliva samples collected from patients with cold-heat complex syndrome ROU before and after treatment. Clinical efficacy parameters including VAS pain scores, TCM syndrome scores, and overall therapeutic evaluations were also assessed. The objective of these measurements was to investigate the efficacy of GXD in treating oral ulcers with cold-heat complex patterns and to compare the clinical outcomes of its decoction and granule forms.

Clinical efficacy analysis revealed significant clinical improvement in patients treated with either GXD or mouthwash combined with vitamin supplementation, demonstrating the therapeutic potential of GXD for cold-heat complex pattern aphthous ulcers. Comparative analysis between the TCM and biomedicine groups showed no statistically significant difference in therapeutic efficacy (*P > 0.05*). These findings suggest that GXD may serve as an alternative therapeutic option to conventional biomedicine for recurrent aphthous ulcers, particularly offering a viable solution for patients with chlorhexidine allergies who face treatment limitations. Statistical analysis showed significant pre-post treatment differences in all three groups (decoction, granules, and biomedicine) for ROU *(P < 0.001*). Further ANOVA comparison of treatment-induced changes revealed no significant differences among groups for EGF, SIgA, IgM, and IgG levels *(P > 0.05*), while EGF and TNF-α showed statistically significant differences *(P < 0.05*). These preliminary findings suggest no significant efficacy difference between GXD decoction and granule formulations, indicating potential clinical equivalence, though large-scale randomized controlled trials are needed for confirmation.

Compared to the decoction, the granule formulation features an instant-dissolve design that reduces patient operational burden. In contrast, preparing traditional decoctions requires a complex process including herbal soaking (30–60 min), boiling (total time ≥60 min), and filtration, which may compromise treatment adherence in today’s fast-paced lifestyle and make it challenging for patients to strictly follow traditional decocting protocols. Additionally, during the study treatment period, a taste evaluation survey was conducted among the 51 enrolled ROU patients. Statistical analysis revealed that 80.4% (41/51) of participants rated the taste experience of GXD below the acceptability threshold, with most explicitly noting the formulation’s pronounced bitter taste profile. This finding suggests that optimizing the palatability of traditional decoctions could represent a critical improvement direction for enhancing patient medication adherence.

While this study provides valuable insights into the relationship between contaminant levels and the clinical efficacy of GXD, several limitations should be considered. First, the sample size was moderate, and all participants were recruited from a single center, which may affect the generalizability of our results. Future multi-center studies with larger cohorts are warranted to validate these findings. Second, although TCM practitioners were calibrated, the differential diagnosis of Cold-Heat Complex Syndrome retains a degree of subjectivity, which is an inherent challenge in TCM clinical research. Third, *a priori* power analysis was conducted during the design stage, based on an assumed effect size (Cohen’s d) of 0.5, with a two-sided α of 0.05 and statistical power of 0.8. The calculated minimum sample size required per group was N = 33. Although the experimental group ultimately enrolled N = 51 participants, the control group comprised only N = 28. Despite observing a statistically significant difference in efficacy (P < 0.05), the preset power requirement (target power = 80%) was not fully met. Therefore, some risk of Type II error (i.e., failure to detect a truly existing difference) remains. The TCM interventions used in this study (such as decoctions) possess distinctive olfactory, gustatory, and visual characteristics that precluded the implementation of blinding for both participants and investigators. Although the control group received conventional biomedicine, significant differences in intervention form between the treatment and control groups made it challenging to develop a placebo with identical sensory attributes, ultimately preventing blinding. Future validation of these findings should be conducted through larger-scale multicenter clinical trials. It is anticipated that subsequent randomized controlled trials with increased sample sizes will further clarify the efficacy differences between GXD granules and decoction formulation.

## Data Availability

The datasets supporting the conclusions of this study are available from the corresponding authors upon reasonable request.
