# Supplementary material for: Comparison of granule and traditional decoction of Gancao Xiexin for cold-heat complex pattern recurrent oral ulcer: a randomized controlled trial
Source: Front Pharmacol. 2026 Jan 5;16:1668985. doi: 10.3389/fphar.2025.1668985 (PMC12813132; doi:10.3389/fphar.2025.1668985)
Supplement: Supplementary file 1 [file Table1.pdf]

Table S1: Statistical Data Comparison of Samples Between the Healthy Group and the Patient Group

Detection Indicators	Healthy Group(n=18)	Patient Group(n=79)	Testing Methods	*t/Z* value	*P* value
EGF（pg/ml)	22.52±1.54	48.98±2.42	*t*-test	44.25	<0.001
IgA（mg/ml)	1.69±0.03	2.24±0.16	*t*-test	14.4	<0.001
SIgA（ug/ml)	143.88±5.53	186.49±2.58	*t*-test	49.3	<0.001
IgM（mg/ml)	1.73±0.03	1.83±0.02	Wilcoxon Mann-Whitney U Test	6.61	<0.001
IgG（mg/ml)	11.88±0.70	14.70±0.71	Wilcoxon Mann-Whitney U Test	6.57	<0.001
TNF-α（pg/ml)	2.89±0.12	4.91±0.13	*t*-test	59.53	<0.001
IFN-γ（pg/ml)	0.27±0.01	0.34±0.01	Wilcoxon Mann-Whitney U Test	6.86	<0.001
IL-1β(pg/ml)	34.41±2.40	80.19±1.69	Wilcoxon Mann-Whitney U Test	6.6	<0.001
IL-17(pg/ml)	0.26±0.01	0.37±0.01	Wilcoxon Mann-Whitney U Test	6.76	<0.001
IL-23(pg/ml)	1.50±0.05	3.01±0.11	Wilcoxon Mann-Whitney U Test	6.6	<0.001
