# Supplementary material for: Comparison of granule and traditional decoction of Gancao Xiexin for cold-heat complex pattern recurrent oral ulcer: a randomized controlled trial
Source: Front Pharmacol. 2026 Jan 5;16:1668985. doi: 10.3389/fphar.2025.1668985 (PMC12813132; doi:10.3389/fphar.2025.1668985)
Supplement: Supplementary file 2 [file Table2.pdf]

Table S2. Comparative Efficacy of GXD Decoction, GXD Granules, and biomedicine Therapy

Biomarker	Group	Pre-treatment (Mean ± SD)	Post-treatment (Mean ± SD)	Intra-group			Difference (Δ)	Inter-group		
				Testing Methods	*t/z*	*P-value*		Testing Methods	F	P-value
EGF	DCT	47.65±2.14	31.51±2.10	*t*-test	42.794	<0.001	16.14±1.89	ANOVA	8.223	<0.001
	GR	49.37±2.53	30.74±1.99	*t*-test	28.866	<0.001	18.63±3.29			
	WM	49.79±2.11	31.07±2.19	*t*-test	42.073	<0.001	18.72±2.35			
IgA	DCT	2.27±0.18	1.74±0.04	*t*-test	14.664	<0.001	0.53±0.18	ANOVA	2.622	0.079
	GR	2.25±0.17	1.77±0.05	*t*-test	16.412	<0.001	0.49±0.15			
	WM	2.19±0.13	1.76±0.06	*t*-test	22.884	<0.001	0.43±0.10			
IgG	DCT	14.65±0.78	12.39±0.61	*t*-test	18.85	<0.001	2.27±0.60	ANOVA	1.091	0.341
	GR	14.75±0.60	12.20±0.53	*t*-test	16.268	<0.001	2.55±0.80			
	WM	14.70±0.75	12.17±0.53	Wilcoxon signed-rank test	4.623	<0.001	2.53±0.85			
IgM	DCT	1.83±0.02	1.74±0.02	*t*-test	15.59	<0.001	0.08±0.03	ANOVA	0.109	0.897
	GR	1.84±0.02	1.75±0.02	*t*-test	15.149	<0.001	0.09±0.03			
	WM	1.83±0.02	1.74±0.02	*t*-test	13.691	<0.001	0.08±0.03			
SIgA	DCT	185.88±2.45	156.16±2.51	*t*-test	38.412	<0.001	29.72±3.87	ANOVA	0.157	0.855
	GR	186.59±2.87	156.89±2.58	*t*-test	38.108	<0.001	29.69±3.97			
	WM	186.95±2.39	157.74±2.48	*t*-test	44.712	<0.001	29.21±3.46			
IL-1β	DCT	79.55±1.65	41.44±1.21	*t*-test	100.213	<0.001	38.11±1.90	ANOVA	2.362	0.101
	GR	80.56±1.73	41.23±2.23	Wilcoxon signed-rank test	4.457	<0.001	39.33±3.18			
	WM	80.41±1.58	41.02±1.02	*t*-test	112.749	<0.001	39.39±1.85			
IL-17	DCT	0.37±0.01	0.26±0.01	Wilcoxon signed-rank test	4.401	<0.001	0.11±0.02	ANOVA	0.229	0.796
	GR	0.37±0.01	0.26±0.01	Wilcoxon signed-rank test	4.477	<0.001	0.11±0.2			
	WM	0.37±0.01	0.26±0.01	Wilcoxon signed-rank test	4.700	<0.001	0.11±0.01			
IL-23	DCT	2.98±0.10	1.80±0.05	Wilcoxon signed-rank test	4.375	<0.001	1.17±0.11	ANOVA	2.774	0.069
	GR	3.05±0.12	1.80±0.05	Wilcoxon signed-rank test	4.459	<0.001	1.25±0.13			
	WM	3.01±0.11	1.81±0.06	Wilcoxon signed-rank test	4.623	<0.001	1.20±0.11			
INF-γ	DCT	0.34±0.01	0.28±0.01	Wilcoxon signed-rank test	4.406	<0.001	0.06±0.01	ANOVA	2.673	0.076
	GR	0.34±0.01	0.27±0.01	Wilcoxon signed-rank test	4.491	<0.001	0.07±0.01			
	WM	0.34±0.01	0.27±0.01	Wilcoxon signed-rank test	4.674	<0.001	0.07±0.01			
TNF-α	DCT	4.85±0.14	3.11±0.10	*t*-test	46.93237	<0.001	1.74±0.19	ANOVA	12.172	<0.001
	GR	4.91±0.12	3.06±0.10	*t*-test	58.80873	<0.001	1.84±0.16			
	WM	4.98±0.11	3.01±0.08	*t*-test	72.1227	<0.001	1.96±0.14			

DCT: GXD Decoction group; GR: GXD Granule group; WM: Biomedicine therapy group
